# Lack of interleukin-33 and its receptor does not prevent calcipotriol-induced atopic dermatitis-like inflammation in mice

**DOI:** 10.1038/s41598-020-63410-z

**Published:** 2020-04-15

**Authors:** Wojciech Pietka, Olav Sundnes, Clara Hammarström, Manuela Zucknick, Denis Khnykin, Guttorm Haraldsen

**Affiliations:** 1 K.G. Jebsen Inflammation Research Centre, University of Oslo and Oslo University Hospital, Oslo, Norway; 2 Department of Pathology, University of Oslo and Oslo University Hospital, Oslo, Norway; 3 Department of Rheumatology, Dermatology and Infectious Diseases, University of Oslo and Oslo University Hospital, Oslo, Norway; 4 Oslo Center for Biostatistics and Epidemiology, Department of Biostatistics, Institute of Basic Medical Sciences, University of Oslo and Oslo University Hospital, Oslo, Norway

**Keywords:** Interleukins, Inflammation, Translational immunology

## Abstract

Current studies addressing the influence of interleukin-33 or its receptor (IL-33R/ST2) on development of atopic dermatitis-like inflammation in mice have reported conflicting results. We compared the response in single- and double-deficient IL-33^−/−^/ST2^−/−^ C57BL/6J BomTac mice in the well-established calcipotriol-induced model of atopic dermatitis. All genotypes (groups of up to 14 mice) developed atopic dermatitis-like inflammation yet we observed no biologically relevant difference between groups in gross anatomy or ear thickness. Moreover, histological examination of skin revealed no differences in mononuclear leukocyte and granulocyte infiltration nor Th2 cytokine levels (IL-4 and IL-13). Finally, skin CD45+ cells and CD3+ cells were found at similar densities across all groups. Our findings indicate that lack of interleukin-33 and its receptor ST2 does not prevent the development of AD-like skin inflammation.

## Introduction

Atopic dermatitis (AD) is a chronic, pruritic skin disease that affects 15–30% of children and 2–10% of adults^1^. The pathogenesis is not fully understood but it is believed to involve multiple factors, including impaired barrier function, immune dysregulation and environmental factors^2^. The result is a local tissue milieu rich in immune cells, including dendritic cells, eosinophils, neutrophils, mast cells, type 2 innate lymphoid cells (ILC2s), and T cells.

Development of atopic lesions can be divided into an acute phase followed by a chronic phase, the former dominated by a Th2 cytokine profile and the latter more complex with simultaneous coexistence of Th1, Th2, Th17 and Th22 immune signatures^3,4^. During the acute phase, Th2 cytokines (such as IL-4, IL-5 and IL-13) are derived from Th2 cells and ILC2s. Indeed, depletion of ILC2s in the skin reduces AD-like inflammation in mice^5,6^.

Interleukin-33 (IL-33) is an IL-1 family member that is constitutively expressed in the nuclei of keratinocytes in murine epidermis^7,8^. The initial observation that IL-33 induced enhanced levels of type 2 cytokines^9^, led to exploration of its potential contribution in atopic dermatitis^10,11^. This was supported by a GWAS showing a link between polymorphisms in the IL-33 receptor- (ST2, officially named interleukin 1 receptor like 1 - IL1RL1) and an increased risk of AD^12^. Moreover, AD patients and mice with AD-like dermatitis have increased IL-33 and ST2 levels^10,13^, and transgenic mice that selectively overexpress IL-33 in keratinocytes develop an AD-like disease^14^. Interestingly, IL-33 together with thymic stromal lymphopoietin (TSLP) and IL-25 is thought to contribute to the activation of ILC2s and Th2 cells, resulting in type 2 inflammation and disruption of epithelial barrier integrity^15^.

Topical treatment of mouse skin with low-calcemic vitamin D3 analogs, such as calcipotriol (MC903), offers a convenient preclinical model of AD^16^ and has been used to assess the role of IL-33 in AD-like disease, albeit yielding contradictory results^5,6,17^. Thus, while Kim *et al*.^6^ reported reduced inflammation in *Tslpr*^−/−^ but not in *Il33*^−/−^ mice, Salimi *et al*.^5^ showed reduced inflammation in *St2*^−/−^ but not in *Tslpr*^−/−^ mutants. We therefore decided to investigate the function of the IL-33/ST2 signaling system by backcrossing single- and double-deficient mutants of ligand and receptor to the same genetic background (C57BL/6J BomTac) and comparing their response to calcipotriol-induced dermatitis. Our results show that neither lack of IL-33 nor of ST2 detectably affect the inflammatory response to calcipotriol.

## Results

### Topical application of calcipotriol results in development of skin inflammation

**Figure 1:**
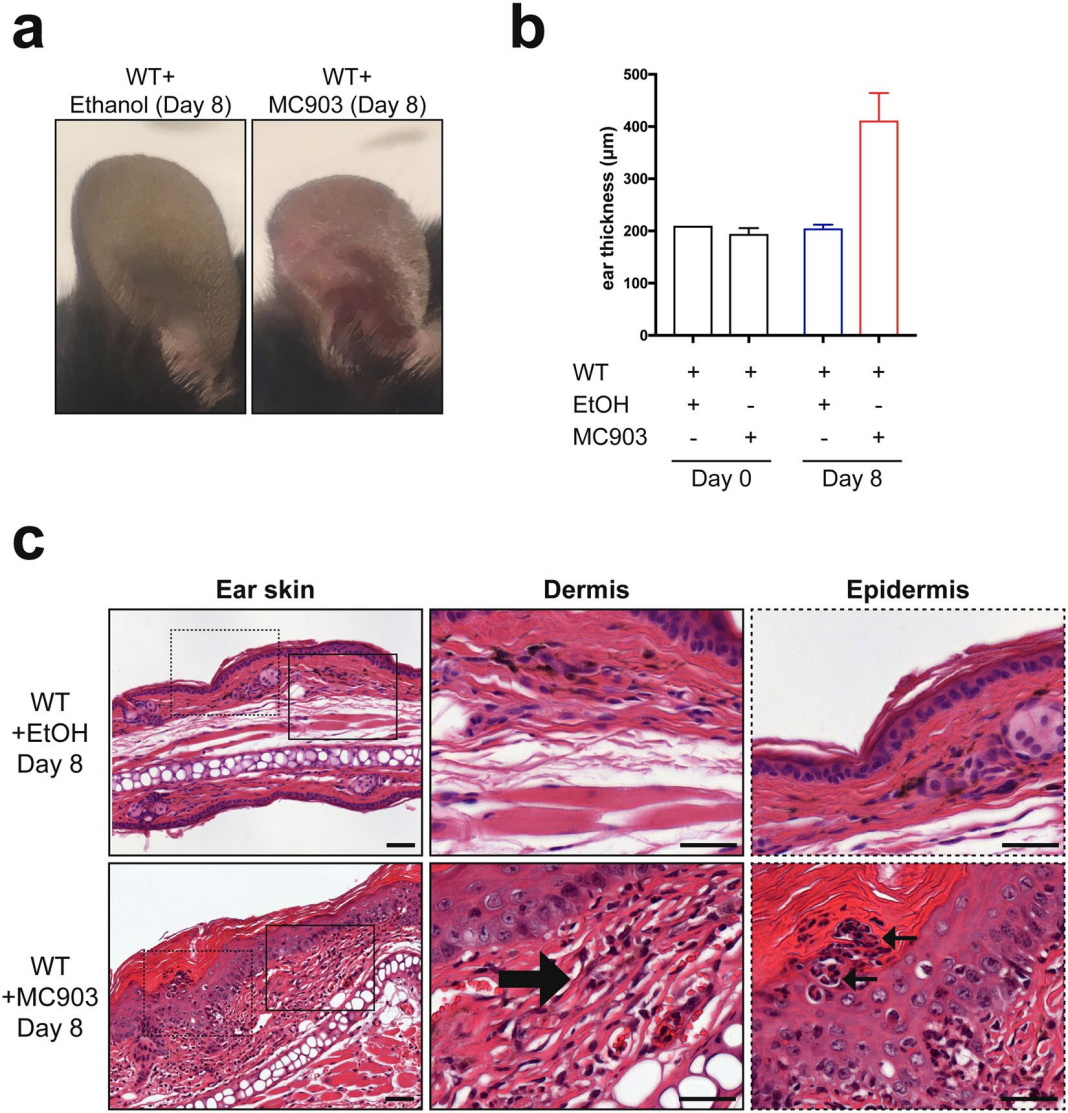
MC903 treatment initiates an AD-like skin inflammation. Appearance of ethanol- and MC903-treated ears at day 8 (**a**). Ear thickness of wild type mice treated with EtOH or MC903, measured on day 0 and day 8 (**b**). Bars are presented as mean + SD; n = 2–7 mice per group (GraphPad Prism v7.0). Hematoxylin and eosin staining of mouse skin treated with EtOH or MC903 analyzed at day 8 (**c**), black arrows show dermal or epidermal mononuclear leukocyte and granulocyte infiltrates. Scale bar 50 μm.

Patients with AD experience chronic relapsing skin inflammation that is preceded by dry, itchy skin^1^. A mouse model that replicates many of the features of AD through daily topical application of low-calcemic vitamin D3 analog – calcipotriol was described by Li *et al*.^16^ Indeed, upon topical calcipotriol treatment, ears of wild type C57BL/6J BomTac mice became dry, thickened and erythematous (Fig. 1a,b). We also observed signs of pruritus in calcipotriol-treated mice as scratching was seen throughout the experiment. Histological characteristics of affected skin included epidermal edema (spongiosis) and a pronounced dermal infiltrate of leukocytes (Fig. 1c).

### IL-33 and ST2 receptor are not required during development of AD-like inflammation

**Figure 2:**
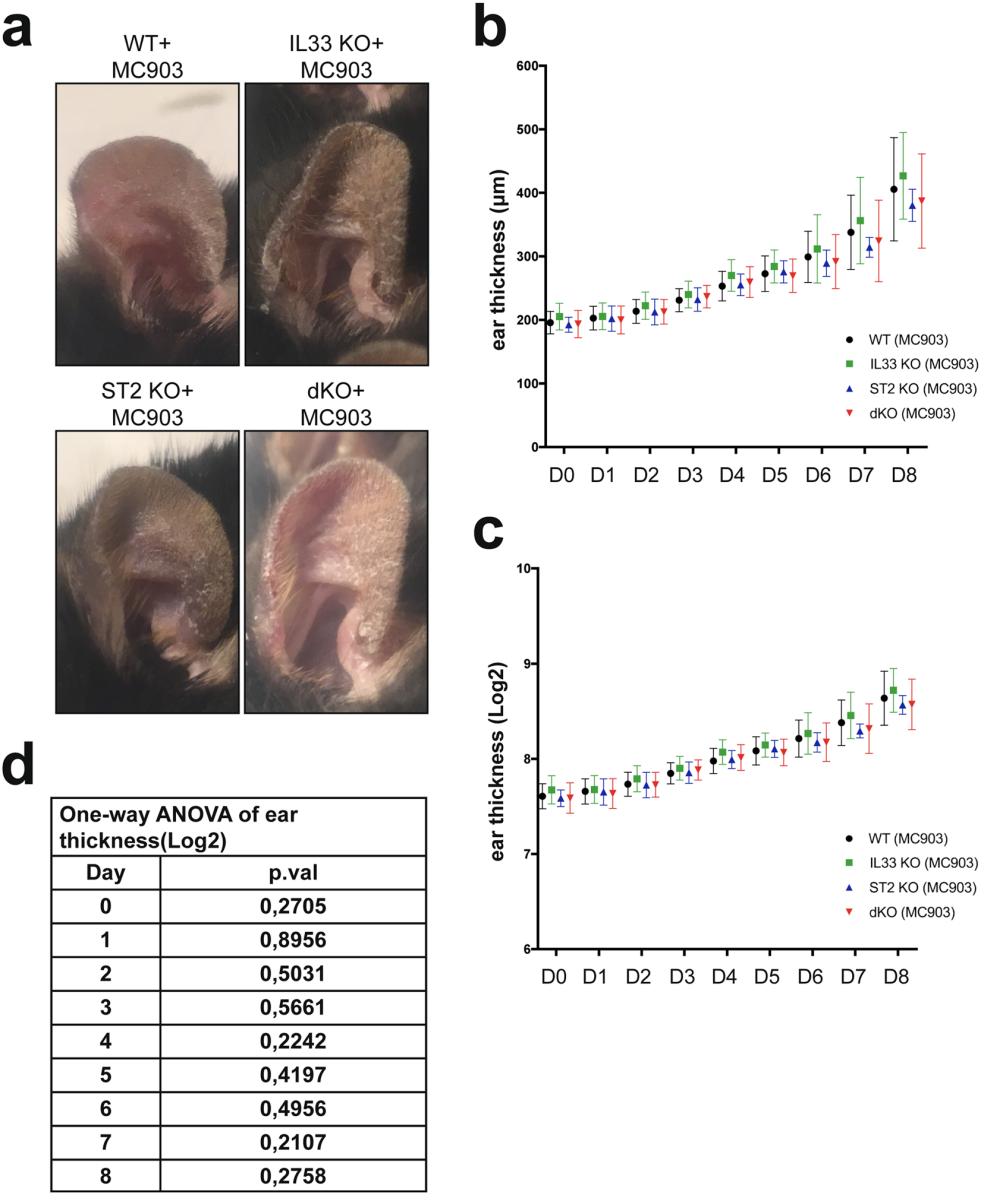
MC903-induced skin inflammation develops similar between mouse groups. Panel (a) represents appearance of MC903-treated mice on day 8 from all four groups. Panels (b,c) show ear thickness measurement throughout the experiment (Day 1–8), representing raw collected data (**b**) and log2-transformed data (**c**). Panel (d) shows one-way ANOVA analysis between groups within specific day performed in GraphPad Prism v7.0. Data are represented as mean ± SD (GraphPad Prism v7.0). Experiments were repeated independently two times; n = 7–14 mice per group per experiment.

To assess the function of IL-33 and its receptor in AD-like inflammation we exposed ears of wild type, *Il33*^−/−^ (IL33 KO), *St2*^−/−^ (ST2 KO) and *Il33*^−/−^*St2*^−/−^ (double-deficient; dKO) mice to daily applications of calcipotriol. The initial ear thickness was similar in each group (day 0) and in the course of the experiment (day 8), mice in all groups developed similar skin phenotypes (Fig. 2a) with a similar increase in ear thickness (Fig. 2b). One observation consistently made through our experiments was that the collected data points were heteroscedastic – variance increased over time. We therefore log2-transformed our ear thickness data in order to achieve homoscedasticity – i.e. make variances constant over time (Fig. 2c), and performed a repeated measures two-way ANOVA and one-way ANOVA analysis. However, we observed no significant difference between the groups throughout the experiment (repeated measures two-way ANOVA, p.val = 0.38) or between groups within a specific day (one-way ANOVA, Fig. 2d). Finally, there was no difference in average ear thickness between groups treated with vehicle at day 0 or day 8 (see Supplementary Fig. S2).

### Calcipotriol treatment results in similar immune cell infiltration of the skin in all mouse groups

**Figure 3:**
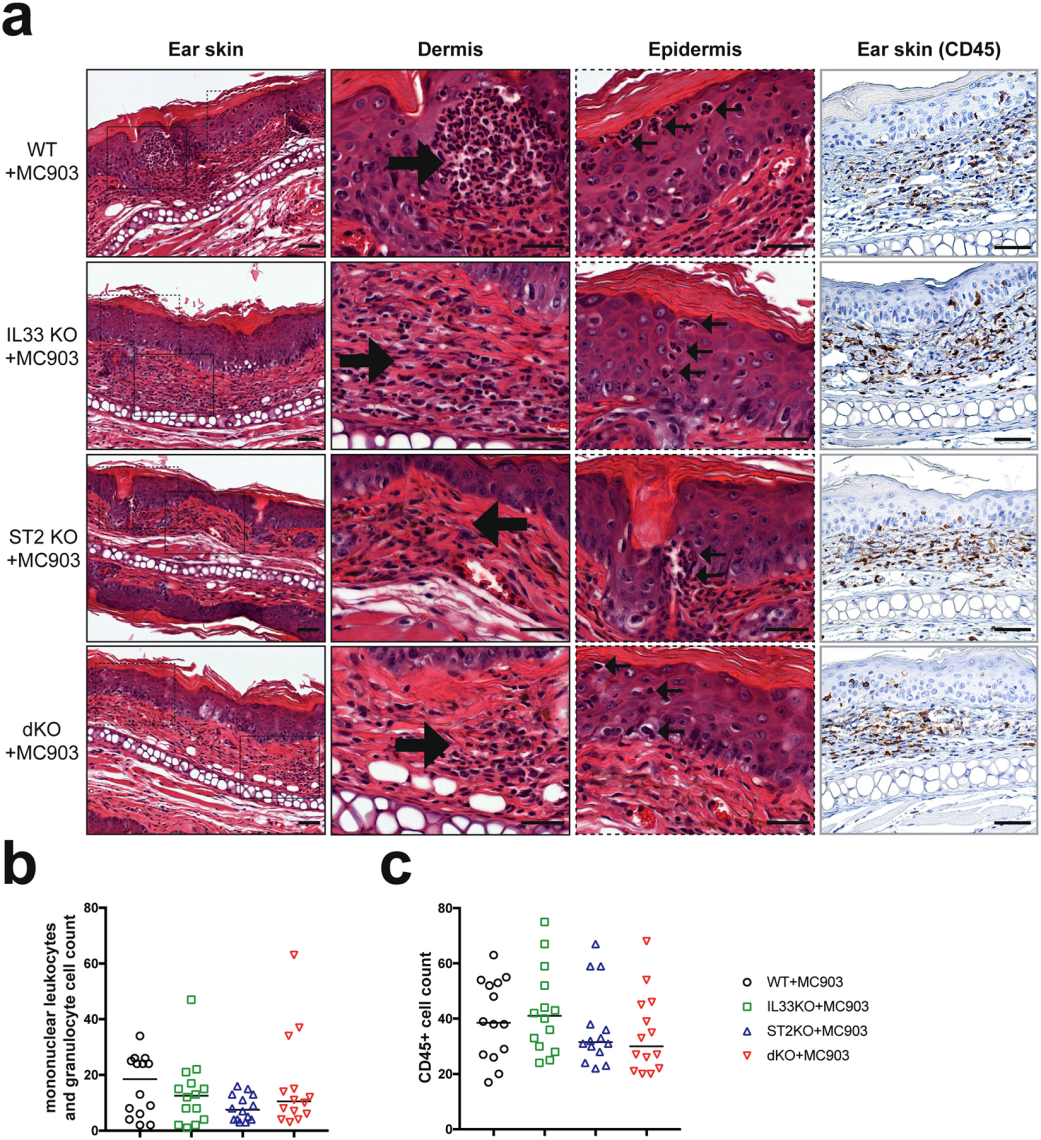
Leukocyte infiltrates are similar across the mouse groups. Panel (a) shows hematoxylin and eosin staining of ear skin tissue; first column represents whole tissue appearance, while the second and third columns show the dermal and epidermal compartment; fourth column shows representative pictures of mouse ears immunostained with CD45 (leukocyte common antigen) antibody. Arrows indicate mononuclear leukocyte and granulocyte infiltrates in the dermis and epidermis. Original magnification x400. Scale bar 50 μm. Panels (b,c) represent quantification of mononuclear leukocytes and granulocytes (**b**), and CD45+ cells (**c**) in the dermal and epidermal compartments (GraphPad Prism v7.0). Data are represented as median value.

At the end of the experiment (day 8) ear tissue was collected, fixed and stained for histological analysis. Consistent with our assessment of ear thickness, we observed and enumerated epidermal and dermal infiltration of mononuclear leukocytes and neutrophils in all groups (Fig. 3a) again observing no significant differences between the groups (Fig. 3b, one-way ANOVA p.val = 0.25). In addition, we determined the number of CD45+ cells present in the skin in each mouse group, observing no significant differences (Fig. 3a,c, one-way ANOVA p.val = 0.48). Finally, we quantified the number of T-cells by CD3+ staining, observing no significant differences between groups (see Supplementary Fig. S3, one-way ANOVA p.val = 0.6).

### Presence or absence of IL-33 or ST2 results in similar Th2 cytokine gene expression

**Figure 4:**
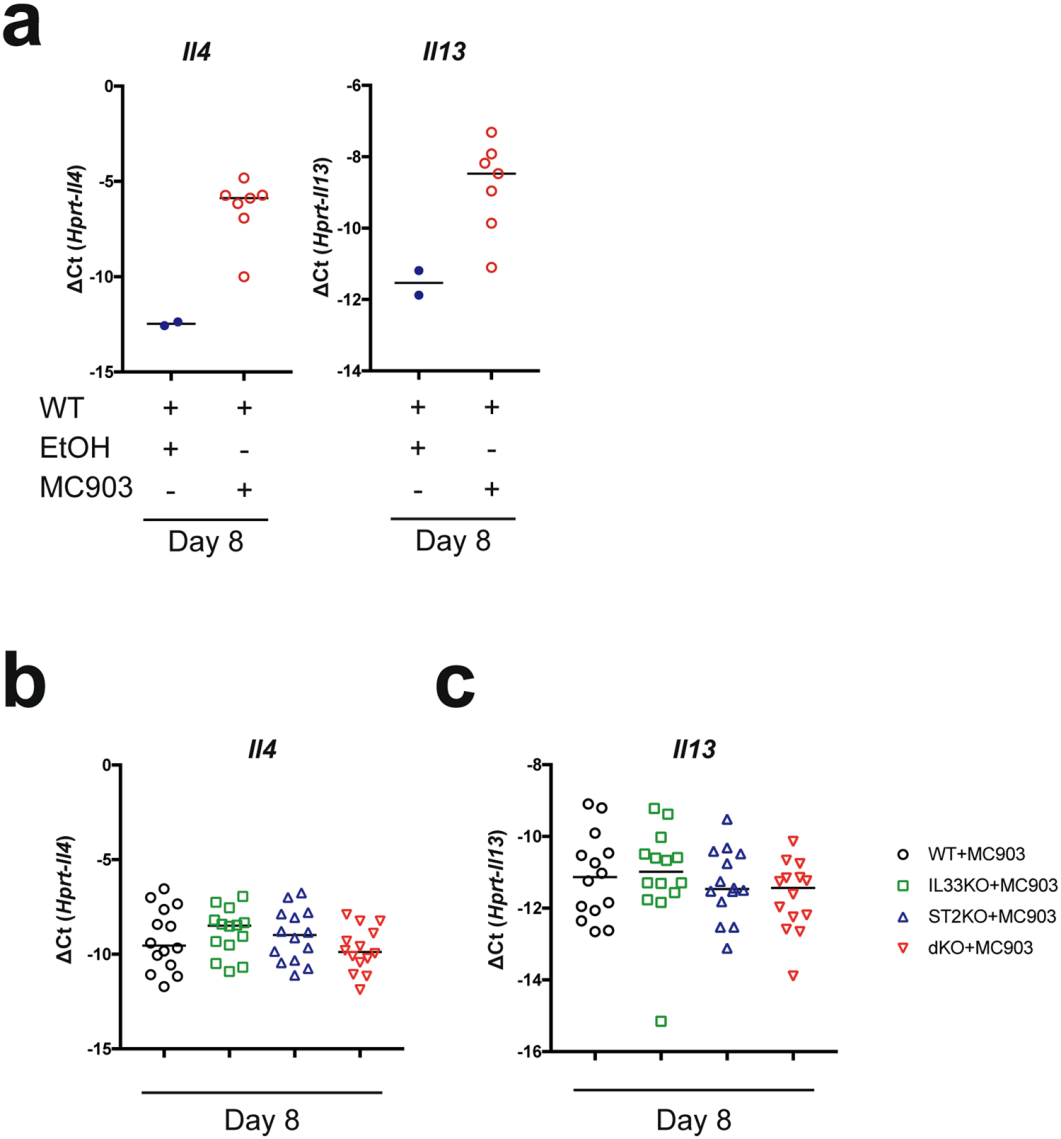
Th2 cytokine expression is similar between MC903-treated mice. Panel (a) represents mRNA expression of *Il4* and *Il13* in MC903-treated or ethanol (EtOH)-treated wild type mice. Panels (b,c) show mRNA expression of *Il4* and *Il13*, in all four mouse groups. Data are represented as median value (GraphPad Prism v7.0).

A Th2 immune signature characterizes the acute phase of AD lesion development^3^ and similarly, transcript levels of IL-4 and IL-13 increase with time in response to calcipotriol treatment when compared to vehicle^16^. In agreement with this report, we found elevated transcript levels at day 8 when comparing the effect of calcipotriol to vehicle in wild type mice (Fig. 4a). We next compared *Il4* and *Il13* induction across all groups at day 8 (Fig. 4b,c), but observed no significant differences between groups (one-way ANOVA, *Il4* p.val = 0.32; *Il13* p.val = 0.51). *Il4* and *Il13* expression in vehicle-treated mice was comparable between groups (see Supplementary Fig. S4).

## Discussion

It has been widely assumed that IL-33 contributes to atopic dermatitis^9,11,14^. This has been supported by a GWAS study showing association between polymorphisms in ST2 receptor and atopic dermatitis^12^ as well as the finding that skin of AD patients and mice with AD-like skin conditions has increased transcript levels of IL-33 and ST2^10,13^. Moreover, in a recent proof-of-concept phase-2a clinical trial an anti-IL-33 antibody (ANB020, etokimab) was successfully used to treat patients with moderate-to-severe adult atopic dermatitis^18^. Yet, IL-33 inhibition by etokimab failed to report efficacy in a follow-up phase-2b clinical trial (NCT03533751) designed to further investigate the safety and efficacy, and the fate of IL-33 inhibition in atopic dermatitis is therefore highly questionable^19^.

On this backdrop, there have been conflicting reports on whether IL-33 is required in the calcipotriol-induced model of AD^5,6,17^. Salimi *et al*.^5^ described ST2-dependent reduction of ear swelling and reduced numbers of ILC2 in the BALB/c strain. Li *et al*.^17^ support these findings by also reporting reduced ear swelling in IL-33-deficient or ST2-deficient mice, yet in BL6 strains of undisclosed origin. By contrast, and in agreement with our results, Kim *et al*.^6^ observed no effect in ILC2 responses or ear thickness in IL-33-deficient C57BL6 Jackson mutants. There are several possible reasons for the clear discrepancies on the role of IL-33 in calcipotriol model of atopic dermatitis.

First, the choice of mouse strain could affect the observed discrepancies. We have used C57BL/6 mice, similar to Kim *et al*.^6^ and Li *et al*.^17^, while Salimi *et al*.^5^. have used BALB/c mice. Although Salimi *et al*.^5^ and Li *et al*.^17^ obtained similar results in BALB/c and BL6 mice, respectively, it has been reported that C57BL/6 substrains differ genetically and even in their phenotype^20,21^. It is therefore possible that our findings in the BomTac strain concur with those obtained in the Jackson strain^6^ but not with those strains used in studies that reported an effect of IL-33 signalling in AD-like disease^5,17^.

Second, genetic drift could be a potential reason for discrepancies between study results. Genetic drift is a tendency of genes to evolve, due to spontaneous mutations that either disappear or become fixed^22^. Small mouse colonies are most vulnerable to genetic drift. Moreover, it is recommended that a mouse colony should be backcrossed to the inbred control strain every 5–10 generations, thus ensuring genetic similarity. In our approach we created double heterozygous *Il33*^+/−^ *St2*^+/−^ animals from already backcrossed mice (WT, *Il33*^−/−^ and *St2*^−/−^). Once the double heterozygous mice were bred together, we were able to obtain all four desired genotypes, which included the wild type, IL33 KO, ST2 KO and dKO that were then kept as separate lines until the 5^th^ generation.

Third, differences in experimental setup could contribute to the observed results. We used, like Kim *et al*.^6^, a calcipotriol dose of 2 nmol dissolved in 20 µl of ethanol, while Salimi *et al*.^5^ used the same amount per site but without reference to vehicle volume. Moreover, Salimi *et al*.^5^ concluded the experiment after day 4, while Kim *et al*.^6^, at day 8. Li *et al*.^17^ used a lower dose but the same concentration while applying calcipotriol (1nmol dissolved in 10 µl ethanol) and concluded the experiment at day 12. Together, these differences may also affect results.

Fourth, use of ligand- (Kim *et al*.^6^, Li *et al*.^17^) or receptor-deficient (Salimi *et al*.^5^, Li *et al*.^17^) mice cannot exclude receptor- or ligand-independent functions of IL-33 or ST2, respectively. Indeed, a possible IL-33-independent function of ST2 was indicated in a model of arthritis^23^, because ST2-deficient mice showed a reduction in disease severity while IL-33-deficient mice were similar to wild type animals. To exclude any ligand or receptor independent effects, our experimental design included, in addition to IL33 KO and ST2 KO, dKO mice.

To test how sensitive our experimental design is in detecting ear thickness changes, we performed a sensitivity analysis of the non-transformed data in G*Power software v3.1^24^. With a sample size of 14 animals per group we were able to detect effect sizes ≥ 1.1 with statistical power of at least 80%. For effect sizes of 1 we still had adequate statistical power of 72%. Note that an effect size of 1 corresponds to the results reported by Salimi *et al*.^5^ for a difference in ear thickness at day 4 between wild type and ST2-deficient mice (where effect size 1 results from a mean difference of 20 μm in ear thickness between the two groups with a standard deviation of approx. 20 μm in both groups). Yet this study – as well as most other previously published studies – does not have sufficient sample size to reliably detect small to moderate group differences. Nevertheless, in a situation where we have also analyzed cytokine profiles and histology, we must allow our imagination to suggest that there is no biologically relevant effect of abrogating IL-33 signaling in AD-like disease.

It also is possible that TSLP and/or IL25 may compensate for the lack of IL-33 signaling in our mutants. This hypothesis can be tested by the generation of double- or triple-deficient mutants lacking two or all cytokines. Such studies deserve future academic attention.

To conclude, the strength of this study relies on the use of animals on a homogenous background and importantly inclusion of double-deficient mutants. The latter allows us to control for either IL-33-independent effects of ST2 or *vice versa*. We believe that inclusion of sufficient methodological detail and a homogenous background of all experimental groups is crucial when performing and interpreting data from such studies.

## Methods

### Animal strains and experiments

C57BL/6JBom mice were purchased from Taconic. IL33 KO animals were crossed with ST2 KO mice and then the double heterozygous mice were crossed again in order to produce double knockout mice. Ear samples were collected from all mice prior to the experiment for total DNA extraction, followed by genomic PCR (see Supplementary Fig. S1). Primer sequences: *Il33* (primer1: 5′-CAGCCTCAGATTTCTCTGTGC-3′, primer2: 5′-TCAGGTTTCTGTGGGATTGA-3′, primer3: 5′-TGTCAACAATGATGCACTGG-3′), *St2* (primer1: 5′-TAACATACGAAACAGAAGCCCA-3′, primer2:5′-CAAGGTGAGATGACAGGAGA-3′, primer3: 5′-CAGATGAGGCACCTAGAGTC-3′) Experiments were repeated independently two times. All animal experiments were performed with institutional approval of Norwegian Food Safety Authority and in accordance with Regional Committee for Medical and Health Research Ethics (REK) and Ministry of Health and Care Services legislation. Experiments were performed on 8–10 week old mice. Both males and females were used for the experiments. Two nmol of calcipotriol from Tocris Bioscience (Bristol, UK) was dissolved in 20 μl of ethanol and administered daily for 8 days to front and back side of both ears. Ear thickness measurements were performed each day using a Kroeplin caliper (Schlüchtern, Germany). In each experiment 7–14 mice per group were used.

### Histology and immunohistochemistry

At the end of the experiment, mice were euthanized and ears collected for histological and PCR analysis. Ear tissue was immediately fixed in 4% paraformaldehyde (PFA) for 24 hours at 4 °C and processed further for paraffin embedding. Thin tissue sections (3μm) were mounted on Superfrost Plus slides (Menzel-Gläser, Braunschweig, Germany) and deparaffinized before hematoxylin/eosin staining. Sections for immunohistochemistry were stained by an automated IHC/ISH slide staining system, Ventana Discovery Ultra from Roche (Basel, Switzerland). For quantification of T-cells, skin biopsies immunostained with CD3 (Rabbit monoclonal anti CD3 clone SP7, abcam Cat# ab16669, dilution 1:50) were assessed. The three fields (at 200x magnification) with highest number of positive cells in each biopsy were selected and the number of positive cells in each field was counted. Anti-CD45 antibody used for immunohistochemistry was from BD Pharmingen (Franklin Lakes, NJ), a Rat anti-Mouse CD45 clone 30-F11, BD (Cat# 550539, dilution 1:50). CD45 detection was performed by Ventana OmniMap anti-Rt HRP (Basel, Switzerland, Cat# 760-4457). For quantification of mononuclear leukocytes and granulocytes, hemotoxylin and eosin-stained sections were assessed. The five fields (at 400x magnification) with highest number of neutrophils were selected and the number of positive cells in each field was counted. Figures were assembled in Adobe InDesign CS6.

### RT-PCR

Ear tissue was isolated on the last day of the experiment, and preserved in RNAlater solution from Sigma-Aldrich (St.Louis, MO) according to the manufacturer’s instructions. RNA was isolated and purified on RNeasy columns from Qiagen (Hilden, Germany). Total RNA was reverse-transcribed by using Oligo(dT) and SuperScript III Reverse Transcriptase (Life Technologies). Gene transcripts were quantified by real-time quantitative PCR (qPCR) using a Stratagene Mx3005P system (Agilent Technologies, Santa Clara, CA). Transcript levels of *Il4* and *Il13* were normalized against transcript levels of *Hprt* and shown on graphs as ΔCt. Primer sequences: *Il4* (forward: 5′-ATGGATGTGCCAAACGTCCT-3′ reverse: 5′-AGCTTATCGATGAATCCAGGCA-3′), *Il13* (forward: 5′-TGCTTGCCTTGGTGGTCTC-3′ reverse: 5′-GGGCTACACAGAACCCGC-3′), *Hprt* (forward: 5′-TGATCAGTCAACGGGGGACA-3′ reverse: 5′-TTCGAGAGGTCCTTTTCACCA-3′).

### Statistical methods

Statistical tests for differences between groups were performed by either repeated measures two-way ANOVA with Tukey’s multiple comparisons tests (across time points) or one-way ANOVA (for individual time points). Statistical tests and estimation of the coefficients of determination were performed with GraphPad Prism software v7.0. All statistical tests were two-sided. Differences with P < 0.05 were considered statistically significant.

Sensitivity analyses for comparisons at one time point between two independent groups with equal variance were performed with G*Power 3.1^24^ assuming a two-sided Student’s t-test and statistical significance level of 0.05.

## Supplementary information

Supplementary Information.
