# Supplementary material for: Lack of interleukin-33 and its receptor does not prevent calcipotriol-induced atopic dermatitis-like inflammation in mice
Source: Sci Rep. 2020 Apr 15;10:6451. doi: 10.1038/s41598-020-63410-z (PMC7160114; doi:10.1038/s41598-020-63410-z)
Supplement: Supplementary file 1 — Supplementary Information. [file 41598_2020_63410_MOESM1_ESM.pdf]

SUPPLEMENTARY INFORMATION

Lack of interleukin-33 and its receptor does not prevent calcipotriol-induced atopic dermatitis-like inflammation in mice.

Wojciech Pietka[1,2], Olav Sundnes[1,2,3], Clara Hammarström[2], Manuela Zucknick[4], Denis Khnykin[1,2] & Guttorm Haraldsen[1,2,*]

[1]K.G.Jebsen Inflammation Research Centre, [2]Department of Pathology, [3]Department of Rheumatology, Dermatology and Infectious Diseases, [4]Oslo Center for Biostatistics and Epidemiology, Department of Biostatistics, Institute of Basic Medical Sciences, University of Oslo and Oslo University Hospital, Norway

*Correspondence: Guttorm Haraldsen, Department of Pathology, Oslo University Hospital Rikshospitalet, PO Box 4950 Nydalen, NO-0424 Oslo, Norway. Phone: (+47) 23 07 14 92 Fax: (+47) 23 07 15 11. E mail: guttorm.haraldsen@medisin.uio.no

Supplementary Figure Legends

Figure S1: Genotyping of IL-33KO, ST2KO and dKO
Panel **a** represents uncropped gel image, with a doted line marking separate genotyping results – upper panel for IL33 and lower panel for ST2. The positions of molecular weight markers are indicated on the left in base pairs.

Figure S2: Ear thickness is comparable in vehicle-treated mice
Panel **a** shows ear thickness of vehicle-treated mice, measured on day 0 and day 8 (GraphPad Prism v7.0).

Figure S3: MC903 treatment leads to similar CD3+ T cell density across groups
Panel **a** shows representative pictures of mouse ears immunostained with CD3 antibody. Arrows indicate CD3 positive cells. Original magnification x200. Scale bar 100 µm. Panel **b** represents quantification of CD3+ cells in epidermal compartments. Data are represented as median value (GraphPad Prism v7.0).

Figure S4: Th2 cytokine expression is comparable between vehicle-treated mice
Panel **a** represents mRNA expression of *Il4* and *Il13* in vehicle-treated mice. Data are represented as ΔCt (GraphPad Prism v7.0).

Fig S1

a

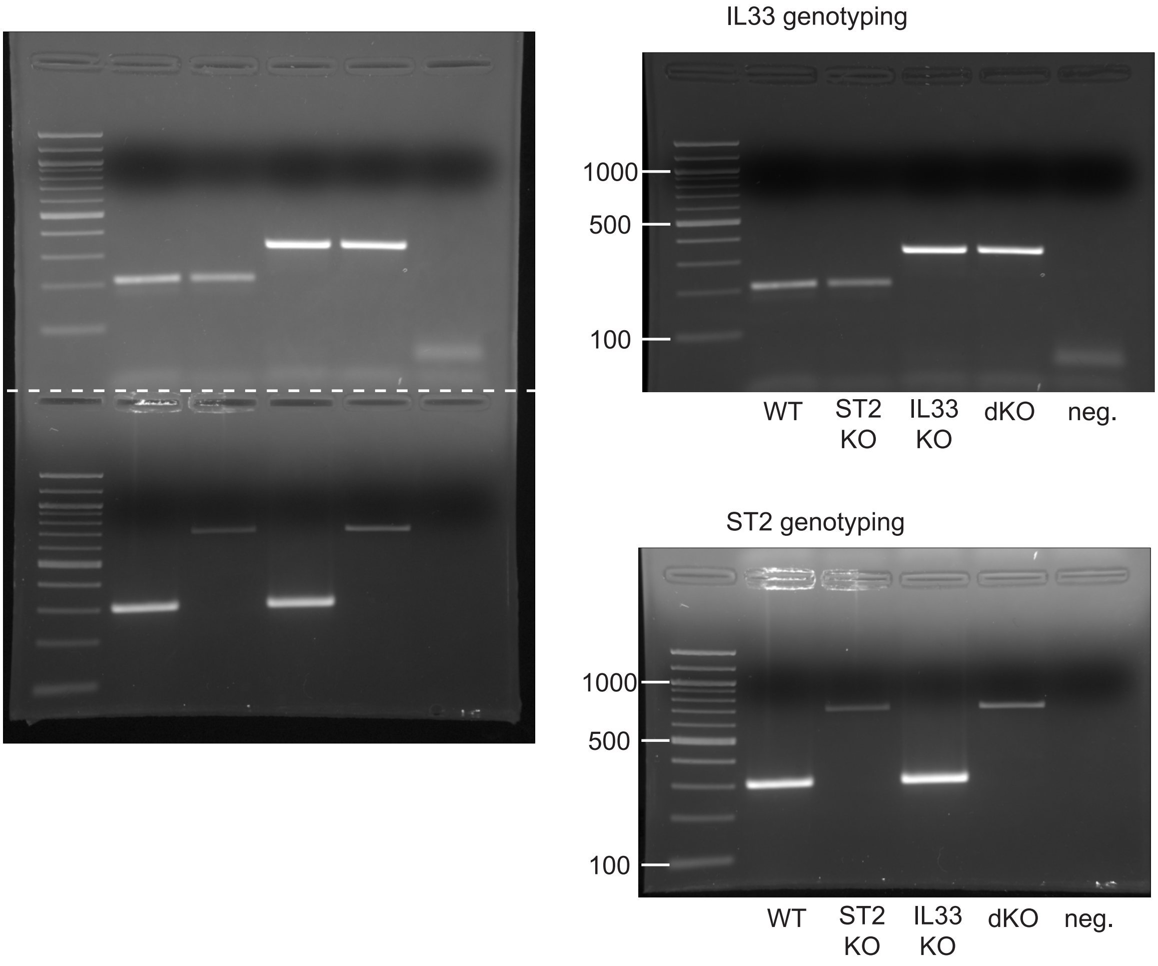
IL33 genotyping
1000
500
100
WT
ST2 KO
IL33 KO
dKO
neg.
ST2 genotyping
1000
500
100
WT
ST2 KO
IL33 KO
dKO
neg.

Fig S2

a

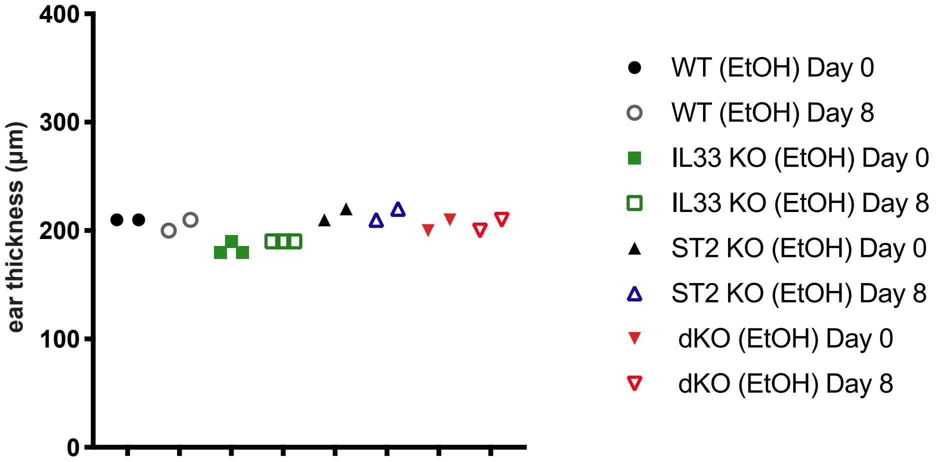
400
300
200
100
0
ear thickness (µm)
WT (EtOH) Day 0
WT (EtOH) Day 8
IL33 KO (EtOH) Day 0
IL33 KO (EtOH) Day 8
ST2 KO (EtOH) Day 0
ST2 KO (EtOH) Day 8
dKO (EtOH) Day 0
dKO (EtOH) Day 8

Fig S3

a

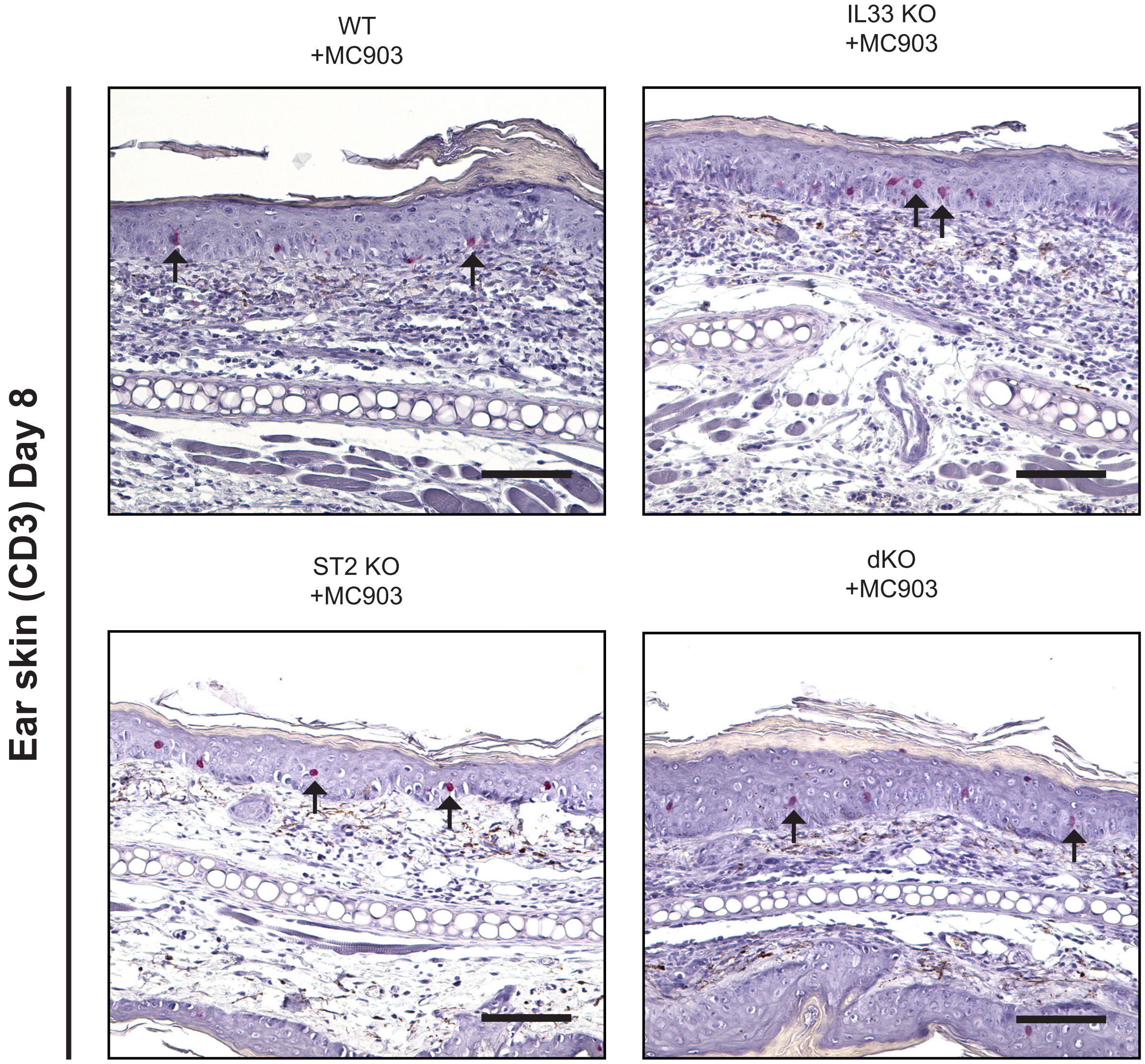
WT
+MC903
IL33 KO
+MC903
ST2 KO
+MC903
dKO
+MC903
Ear skin (CD3) Day 8

b

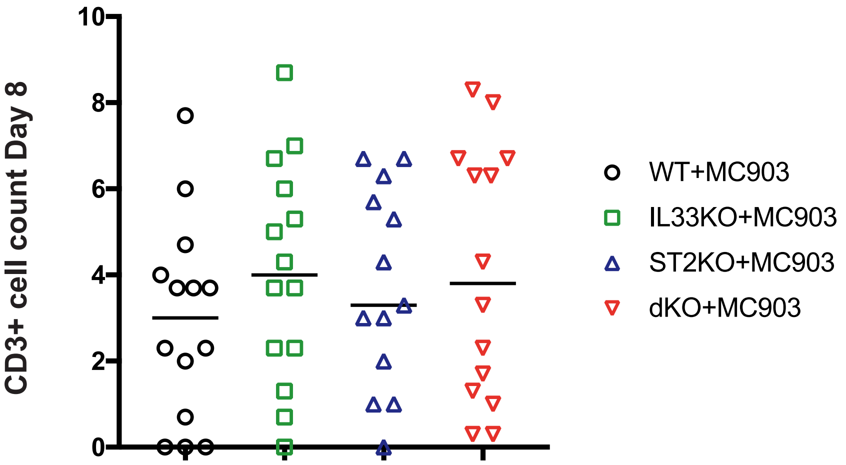
CD3+ cell count Day 8
10
8
6
4
2
0
WT+MC903
IL33KO+MC903
ST2KO+MC903
dKO+MC903

Fig S4

a

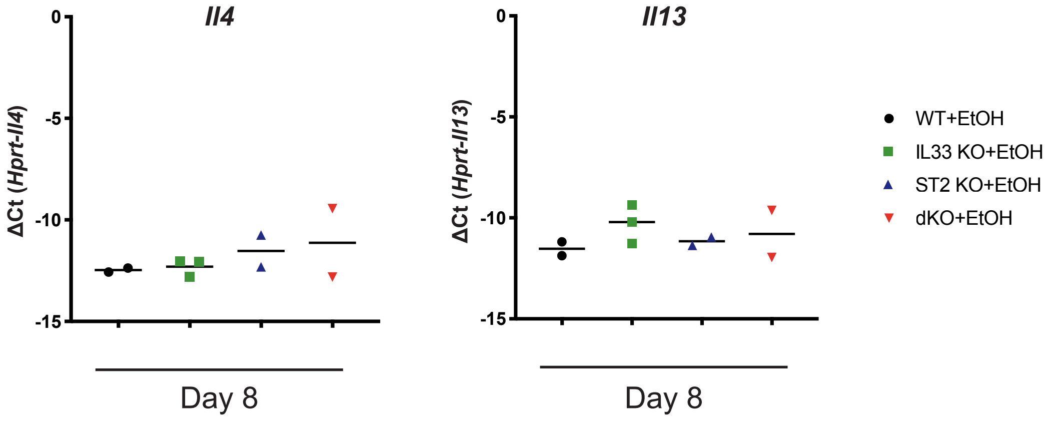
Il4
Il13
ΔCt (Hprt-Il4)
ΔCt (Hprt-Il13)
0
-5
-10
-15
Day 8
Day 8
WT+EtOH
IL33 KO+EtOH
ST2 KO+EtOH
dKO+EtOH
